# Supplementary material for: Selective STAT3 Allosteric Inhibitors HCB-5300 and HCB-5400 Alleviate Dextran Sulfate Sodium-Induced Ulcerative Colitis in Mice
Source: Int J Mol Sci. 2025 Dec 12;26(24):11981. doi: 10.3390/ijms262411981 (PMC12733274; doi:10.3390/ijms262411981)
Supplement: Supplementary file 1 [file ijms-26-11981-s001.zip › ijms-4014355-supplementary.pdf]

Selective STAT3 Allosteric Inhibitors HCB-5300 and HCB-5400 Alleviate Dextran Sulfate Sodium-Induced Ulcerative Colitis in Mice

Wook-Young Baek [1], Ji-Won Kim [1], So-Won Park [2], Nan Kim [3], Sun-Gyo Lim [4] and Chang-Hee Suh [1,2,*]

Supplementary table and figures

		Marketed therapies (literature)		STAT3 T-cell KO	Preclinical-stage candidates			
		Immunosuppressants	JAK inhibitors		GPR40/ STAT3	STAT3	Huchembio	
		Azathioprine	Tofacitinib		TAK-875	TTI-101	HCB-5300	HCB-5400
mpk (mg/kg)		10 (P.O.)	15 (P.O.)		20/30 (P.O.)	100 (I.P.)	25 (P.O.)	**12.5 (P.O.)**
FREQUENCY		Daily (18 days)	3/day (3 days)		Daily (8 days)	Daily (7 days)	Daily (7 days)	Daily (7 days)
Recovery (%)	Colon length		70	35	80/55	90	30	**75**
	Colon inflammation score	-10				90	**45**	**60**
	Histological score	30		40	35/60		**40**	**50**
	Mucosal damage	30					**65**	**90**
	DAI score		50		50/70	100(?)	40	40
Mice (n/group)		6	8		6	9	5	5

Supplementary Table S1. Literature-based benchmark analysis of therapeutic efficacy of HCB-5300 and HCB-5400 compared with representative standard therapies in DSS-induced colitis models. Literature-based benchmark analysis comparing the effects of HCB-5300 and HCB-5400 on body weight, DAI, colon length, and histology with representative data reported for Azathioprine, Tofacitinib, TAK-875, and the STAT3 inhibitor TTI-101 in DSS-induced colitis models.

A

Parameter	Unit	HCB-5300				
		1 mg/kg	5 mg/kg	10 mg/kg	20 mg/kg	50 mg/kg
t1/2	h	0.00	3.95	2.64	3.87	4.93
Tmax	h	0.50	0.50	0.50	0.25	0.25
Cmax	ng/ml	3	9	45	120	327
AUC 0-t	ng/ml*h	1	16	54	168	889
AUC 0-inf_obs	ng/ml*h	0	22	63	222	909
MRT 0-inf_obs	h	0.00	5.57	3.38	4.91	5.30
Vz/F_obs	(mg)/(ng/ml)	0.000	1.309	0.624	0.498	0.459
Cl/F_obs	(mg)/(ng/ml)/h	0.000	0.228	0.161	0.093	0.062

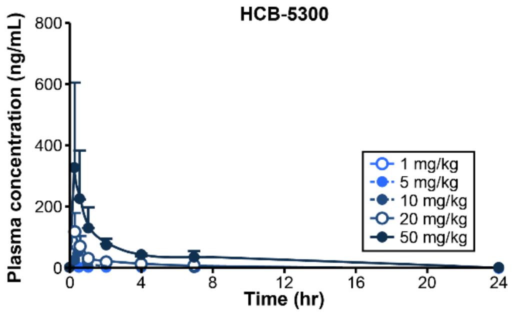

B

Parameter	G1_IV 1 mg/kg	G2_PO 10 mg/kg
AUC(last)_ng/mL·h	411.2	7538.5
AUC(inf)_ng/mL·h	532.2	7544.0
MRT(inf)_h	5.5	5.3
Cmax_ng/mL[2)]	74.8	791.4
Tmax_h	2.0	0.5
CL(obs)_mL/min/kg_PO	31.3	22.1
Vss(obs)_L/kg_IV	10.4	NC[3)]
Vz(obs)_L/kg_PO	9.6	4.1
C0_ng/mL	25.8	NC
$t_{1/2}$_h	3.5	2.1
**Bioavailability (%)**		**183.3**

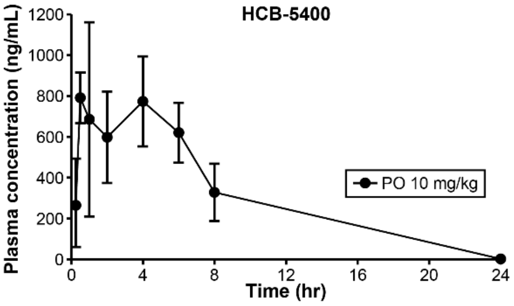

Supplementary Figure S1. PK profiles of HCB-5300 and HCB-5400. (a) Plasma concentration–time profile of HCB-5300. (b) Plasma concentration–time profile of HCB-5400.

A

		HCB-5300		
		0.1 μM	1 μM	10 μM
Kinase (% activity)	JAK2(h)	95	103	128
	JAK3(h)	136	122	78

B

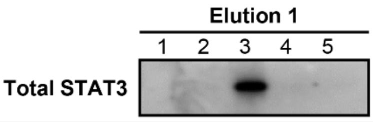

	1	2	3	4	5
Biotin (μM)	10	0	0	0	0
HCB-5300_Biotin-1 (μM)	0	10	0	10	0
HCB-5300_Biotin-2 (μM)	0	0	10	0	10
HCB-5300 (μM)	0	0	0	100	100

Supplementary Figure S2. Mechanism of action (MOA) and proof of concept (POC) of the selective STAT3 allosteric inhibitor HCB-5300. (a) JAK2/JAK3 kinase assays showing that HCB-5300 does not directly inhibit JAK kinases at pharmacologically relevant concentrations, supporting target selectivity at the STAT3 level.
(b) Biotin pull-down assay using biotinylated HCB series compounds showing specific physical interaction with STAT3 protein, further validating these compounds as selective allosteric STAT3 inhibitors.
